# Group Psychological Therapy Program in Adult Patients with Congenital Heart Disease and Anxious–Depressive Symptoms

**DOI:** 10.3390/medicina61010090

**Published:** 2025-01-07

**Authors:** Efrén Martínez-Quintana, Karen Codana-Alcántara, Hector M. Montesdeoca-Naranjo, Marta Isabel García-Suárez, María Pino Fleitas-Álvarez, María Alcántara-Castellano, Alejandro Ruiz-Castellano, Ana González-Isasi, Fayna Rodríguez-González, Esperanza Bosch-Casañas

**Affiliations:** 1 Cardiology Service, Complejo Hospitalario Universitario Insular-Materno Infantil, Avenida Marítima del Sur, s/n, 35016 Las Palmas de Gran Canaria, Spain; 2 Department of Medical and Surgical Sciences, Faculty of Health Sciences, Universidad de Las Palmas de Gran Canaria, Avenida, Marítima del Sur s/n, 35016 Las Palmas de Gran Canaria, Spain; 3 Clinical Psychology Unit, Psychiatry Service, Complejo Hospitalario Universitario Insular-Materno Infantil, Avenida Marítima del Sur, s/n, 35016 Las Palmas de Gran Canaria, Spain; 4 Hospital Universitario de Gran Canaria Dr. Negrín, Barranco de la Ballena, s/n, 35010 Las Palmas de Gran Canaria, Spain; 5 Psychiatry Service, Complejo Hospitalario Universitario Insular-Materno Infantil, Avenida Marítima del Sur, s/n, 35016 Las Palmas de Gran Canaria, Spain

**Keywords:** congenital heart disease, anxiety, depression, adult, psychological

## Abstract

*Introduction*: Anxiety and depression are significant mental health concerns for individuals with congenital heart disease (CHD). As group therapy has proven to be a valuable and effective treatment option for managing anxiety and depression, the aim of this study was to determine its effects on patients with CHD and anxious–depressive symptoms. *Methods*: We used non-pharmacological psychological group intervention, of six weekly sessions of 90 min each, administered by trained personnel, in adult patients with CHD. Measurement tools included quality of life (Euro quality of life-5D questionnaire), self-esteem (Rosenberg Self-Esteem Scale), anxiety (State–Trait Anxiety Inventory), depression (Beck Depression Inventory-II), and satisfaction surveys. *Results*: A total of 18 out of 21 CHD patients (mean age 35.8 ± 9.0 years old and 13 (72%) females) completed the program. According to CHD complexity, five (28%) patients had mild, six (33%) moderate, and seven (38%) great defects. Patients with CHD scored significantly higher in the Euro quality of life visual analogue scale (7.83 ± 1.4 vs. 7.14 ± 1.6, *p* = 0.012) and lower in the Beck Depression Inventory-II (12.3 ± 10.9 vs. 18.1 ± 12.1, *p* = 0.003) post-program than pre-intervention. Meanwhile, the Rosenberg Self-Esteem Scale score was close to reaching statistical significance (27.4 ± 6.0 vs. 25.1 ± 5.4, *p* = 0.051), while the State–Trait Anxiety Inventory did not. Finally, participants scored high in the satisfaction questionnaire at the end of the sessions, on a scale from 0 to 3, especially in the questions related to feeling comfortable with others (2.5 ± 0.6), recommending the program (2.3 ± 0.6), or being willing to attend future sessions (2.6 ± 0.8). *Conclusions*: Group psychological therapy proved to be a useful tool to reduce depressive symptomatology after a 6-week program, providing a comfortable environment to patients with CHD.

## 1. Introduction

Psychological problems are prevalent among individuals with chronic diseases, significantly impacting their overall quality of life and disease management. Chronic conditions, such as diabetes, heart disease, and autoimmune disorders, often lead to persistent stress, anxiety, and depression due to the ongoing nature of the illness, the burden of treatment regimens, and the uncertainty regarding disease progression. These psychological challenges may exacerbate physical symptoms, with psychological distress leading to maladaptive coping strategies, poor adherence to treatment, or a worse clinical outcome [1].

Similarly, chronic heart disease not only affects physical health but also has significant individual psychological complications. The fear of potential cardiovascular events can create a constant state of worry, contributing to heightened stress levels. Additionally, the limitations imposed by the disease, such as reduced physical activity and the need for ongoing medical care, can lead to feelings of helplessness and frustration [2]. Also, on a social level, chronic heart disease may lead to significant complications in interpersonal relationships and social interactions. Patients may withdraw from social activities due to physical limitations or fear of exacerbating their condition, leading to isolation and loneliness. This social withdrawal can strain relationships with family and friends, who may struggle to understand the emotional and physical challenges faced by the patient [3].

Congenital heart disease (CHD) refers to problems with the heart structure that are present at birth. It is a large, rapidly emerging global problem with an incidence that varies from about 4/1000 to 50/1000 live births [4,5]. As a result of advances in diagnosis and treatment in recent years, more than 90% of these patients reach adulthood [6]. Despite this greater survival, adults with CHD frequently require surgical and/or percutaneous interventions, the implantation of devices such as cardiac prostheses, pacemakers, or defibrillators, or oral anticoagulation. Alongside this is the fact that patients with CHD may also present additional difficulties [7] such as limitations in physical exercise, speech or language disorders, concentration difficulties, or antisocial behavior, which may lead to a higher prevalence of attention deficit disorders, anxiety, or depression [8,9]. In fact, one in three adults with CHD have mood or anxiety disorders [10,11,12,13], higher than what is found in the general population [14]. Therefore, the mental study of these patients is essential, proposing strategies for early diagnosis and adequate control.

Group psychological interventions for patients with CHD have emerged as a valuable approach to address the unique emotional and psychological challenges faced by this population. These interventions provide a supportive environment where individuals can share their experiences, reduce feelings of isolation, and develop coping strategies in a collective setting. By fostering peer connections and facilitating open discussions about the emotional impact of living with CHD, these interventions not only empower patients but also promote resilience and adaptive coping mechanisms [15]. One commonly used tool in group therapy is mindfulness-based interventions, which encourage participants to develop greater awareness of their thoughts and feelings in the present moment. Techniques such as guided meditation, breathing exercises, and body scans can be introduced during sessions to help individuals manage their anxiety and depressive symptoms. By practicing mindfulness together, group members can learn to observe their thoughts without judgment, reducing the impact of negative emotions and stressors in their daily lives [16]. Also, Jacobson’s Progressive Muscle Relaxation can be a powerful tool for individuals dealing with anxiety and depression as it helps to relax different muscle groups in the body, which favors physical relaxation and reduces stress [17]. 

By providing a safe and supportive environment, group therapy intervention may help individuals understand and manage their problems through identifying and changing negative thought patterns and behaviors [18]. Unfortunately, most adults with CHD and depression or anxiety do not receive mental health treatment. Moreover, during the coronavirus pandemic, the prevalence of stress, anxiety, or depression increased compared to the pre-pandemic period among patients with CHD, with most of these patients undertreated [19]. In this context, Canadian, American, and European guidelines for the care of adults with CHD emphasize the importance of psychological care for this subgroup of cardiac patients [20,21,22].

The aim of this study is to carry out a non-pharmacological psychological group intervention, administered by trained personnel, in adult patients with CHD, with the main objective of determining its effects on anxiety and depression through questionnaires. As a secondary objective, quality of life, self-esteem, compliance with attending the scheduled sessions, and final satisfaction with the group therapy program were also determined.

## 2. Methods

### 2.1. Participants

Patients were obtained from a single outpatient CHD unit through face-to-face interview, email, and/or telephone calls. Phone calls were carried out on people with anxious–depressive symptoms determined in previous studies [13]. The emails included a cover letter, signed by those responsible for the study, and a document with the topics of the sessions, dates, the professional in charge, to whom the program was directed, and the objectives the group program wanted to achieve. Likewise, patients who wished to participate after the telephone call or the face-to-face interview were sent an email with this information.

Patients were eligible if they (1) had a CHD verified by imaging tests, (2) were 18 years or older, (3) had clinically associated anxiety–depression symptoms determined by the psychology team, (4) could read and complete the consent form and questionnaires, (5) were willing to participate in a working therapy group, (6) did not have surgery planned during study participation or in the year prior to the inclusion in the study, and (7) did not have comorbidity problems which limited their life expectancy. Exclusion criteria included (1) psychotherapy at the time of study participation; (2) significant cognitive impairment; and (3) patients who did not want to participate in the study or did not sign the written consent form. During follow-up, none of the patients in the study underwent psychological therapy, nor were any changes made to the patient’s usual treatment. The study protocol was approved by the Ethics Committee of our hospital and is in accordance with the Declaration of Helsinki for experiments with human beings.

The patient inclusion period was carried out between October 2023 and February 2024. In total, 135 consecutive clinical interviews were carried out during routine check-ups, 413 emails were sent, and 35 telephone calls were made. Telephone calls were specifically directed to patients who had reported having previous anxiety–depressive symptoms. A total of 27 patients confirmed their wishes to participate in the study: 14 patients were recruited through email sending, 11 after contacting them by telephone, and 2 through a direct interview during routine consultation. Finally, 21 out 27 patients attended the first meeting and 18 out of 21 patients finished the group psychological therapy program and completed both the initial and final surveys.

### 2.2. Data Collection

The patients were interviewed, and data were obtained after verification of the hospital clinical history. Demographic and clinical data included age, sex, type of CHD, New York Heart Association (NYHA) functional class, cardiovascular risk factors (arterial hypertension, diabetes mellitus, dyslipidemia, or smoking habit) [23], the consumption of illicit drugs or alcohol abuse, having a pacemaker or an implantable automatic defibrillator, having previous psychiatric pathology, being under treatment for anxiety or depression, the number of previous cardiac surgeries, main current work activity (employed, retired, stay-at-home, students, unemployed, and others), and educational level (elementary education, primary education, secondary education, and university studies). CHD was classified according to anatomical complexity into simple, moderate, and great [24].

### 2.3. Interventions

Weekly sessions of 90 min each, taught by psychologists, psychiatrists, and cardiologists from our hospital, were carried out over 6 consecutive weeks. All participants received written and/or online information to achieve greater adherence and reinforcement to the sessions. Attendance and completion of assigned tasks were monitored. Surveys, carried out at the beginning and at the end of group therapy, were conducted by the psychiatry/psychology service with the objective of determining the existence of anxious–depressive symptoms at the beginning and end of the program. All patients signed a written informed consent form before being included in the study.

### 2.4. Psychological Program and Feasibility of the Group Intervention

We developed a specific program for our population of congenital patients based on a previous group psychosocial intervention aimed at improving the psychosocial functioning, quality of life, and resilience of adults with CHD [15]. This previous work focused on coping with CHD problems, providing a group setting to discuss a variety of important topics such as managing conflicts, social expectations, or advanced care planning. Before starting the psychological program, our patients were asked what aspects they thought should be included during the intervention, expressing a strong interest in areas such as stress management, coping with heart disease, addressing mood and anxiety disorders, and sharing emotional experiences with other individuals affected by CHD. We also included, after the internal discussion of the work team, topics such as physical limitations patients with CHD may have, the recommended diet and exercise they should follow and carry out, the prevalence of workplace harassment among patients with CHD [25], a topic of which we are particularly sensitive, or the risk of suicide seen in patients with chronic diseases [26].

The group intervention, carried out by Kovacs et al. [27] during eight 90 min weekly sessions among patients with CHD, obtained feasibility in the following domains: (i) process (e.g., recruitment or participation), (ii) resources (time and budget), (iii) management (human and data management), (iv) scientific outcomes (safety and treatment fidelity), and (v) intervention acceptability (adherence and acceptability by patients and healthcare team). Table 1 shows the program per-weekly session and the time allocation for each of the sessions carried out in our patients with CHD.

### 2.5. Measurement Tools

#### 2.5.1. EuroQol-5D (EQ-5D)

EQ-5D is a widely used instrument which evaluates the generic quality of life that was developed in Europe; it essentially consists of 2 pages: the EQ-5D descriptive system and the EQ visual analogue scale (EQ VAS). The descriptive system comprises five dimensions: mobility, self-care, usual activities, pain/discomfort, and anxiety/depression. Each dimension has 5 levels: no problems, slight problems, moderate problems, severe problems, and extreme problems, scoring from 0 to 5. The EQ VAS records the patient’s self-rated health on a vertical visual analogue scale where the endpoints are labeled ‘The best health you can image’ and ‘The worst health you can image’. The VAS can be used as a quantitative measure of health outcomes that reflects the patient’s own judgment [28]. The EuroQol-5D (EQ-5D) questionnaire is widely used to measure health-related quality of life, and its reliability has been established in various studies. The reliability coefficients, often reported as Cronbach’s alpha, typically range from 0.70 to 0.90, indicating good to excellent internal consistency. For example, a study by Herdman et al. [29] reported Cronbach’s alpha of 0.76 for the EQ-5D in a general population sample.

#### 2.5.2. Global Assessment of Functioning (GAF)

The GAF scale reflects the degree to which a person’s current symptoms affect their psychological, social, and occupational functioning on a range from 0 to 100. The GAF scale is a tool used to assess an individual’s overall level of functioning, particularly in the context of mental health. The reliability coefficients for the GAF have been reported in various studies, with inter-rater reliability typically ranging from moderate to good, often reported with values around 0.60 to 0.90, depending on the context and population being studied [30]. The assessment of the symptom level of GAF focuses on the severity of the symptoms, extent, duration, and the consequences this has for the person’s self-perception and quality of life. A score of 1 represents the worst imaginable level of symptom severity and impairment of psychosocial functioning, and a score of 100 represents the, hypothetically, most optimal level [31].

#### 2.5.3. Rosenberg Self-Esteem Scale (RSES)

The purpose of the 10-item RSES is to measure self-esteem. Originally, the measure was designed to measure the self-esteem of high school students. However, since its development, the scale has been used with a variety of groups, including adults, with norms available for many of those groups. The RSES is a widely used tool for measuring self-esteem. The reliability coefficients for the RSES are generally high, with Cronbach’s alpha values typically reported in the range of 0.77 to 0.88, indicating good internal consistency. Test–retest reliability is also favorable, often reported at around 0.85, suggesting that the scale produces stable results over time [32]. The scale can be scored by totaling the individual 4-point items after reverse-scoring the negatively worded items. The total score may range from 10 to 40, being sometimes divided into three levels: low (10–25): feelings of incompetence, inadequacy, and difficulty facing life’s challenges; medium (26–29): fluctuating between feelings of approval and rejection; and high (30–40): self-judgment of value, confidence, and competence. The RSES correlates significantly with other measures of self-esteem, including the Coopersmith Self-Esteem Inventory. In addition, the RSES correlates in the predicted direction with measures of depression and anxiety [33,34].

#### 2.5.4. State–Trait Anxiety Inventory (STAI)

To measure the level of anxiety, the State-Trait Anxiety Inventory (STAI) questionnaire was used [35]. This is a 40-item self-assessment questionnaire consisting of two subscales that provide separate measures of two components of anxiety: state and trait anxiety. The first twenty items measure situational or state anxiety (STAI-S), asking people to describe how they generally feel, and the second twenty items measure underlying or trait anxiety (STAI-T), requiring subjects to indicate how they feel at a particular moment in time. The STAI is a well-established tool for measuring anxiety levels. The reliability coefficients for the STAI questionnaire are generally strong. For the STAI-S, Cronbach’s alpha values typically range from 0.86 to 0.95, while for the STAI-T, they usually fall between 0.83 and 0.90. These values indicate good internal consistency for both scales [36]. Each question is rated on a 4-point scale (not at all, somewhat, moderately so, very much so). The range of possible scores is from a minimum score of 20 to a maximum score of 80 on both the STAI-T and STAI-S subscales. STAI scores are commonly classified as “no or low anxiety” (20–37), “moderate anxiety” (38–44), and “high anxiety” (45–80).

#### 2.5.5. Beck Depression Inventory-II (BDI-II)

The evaluation of depression levels was conducted through the Beck Depression Inventory-II (BDI-II). The BDI-II has demonstrated strong reliability coefficients. The internal consistency of the BDI-II is typically reported with Cronbach’s alpha values ranging from 0.91 to 0.94, indicating excellent reliability for the instrument [37]. The Spanish adaptation of the BDI-II, a self-administered tool designed to gauge depressive symptoms, was employed for this purpose [38]. The inventory comprises 21 items, with each item being assessed on a scale ranging from 0 to 3 points. This scoring method yields a total score that spans from 0 to 63. In individuals with a psychological disorder, a score between 0 and 13 may be considered indicative of minimal depressive symptomatology, 14–19 as mild, 20–28 as moderate, and 29–63 as severe, as established by Beck et al. [37].

#### 2.5.6. Satisfaction Questionnaire

A patient satisfaction survey, carried out by the participating team, was used to collect feedback from patients to measure their satisfaction with the quality and usefulness of the program at the end of the sessions. It consisted of 10 questions. The first two questions, that referred to the fulfillment of activities carried out online and at home, scored from 0 to 3 as never, ever, frequently, or always. The rest of the questions (3 to 10), that referred to the general satisfaction of the program, scored from 0 to 3 as not suitable at all, partially suitable, adequate, or very suitable.

### 2.6. Statistical Analysis

Continuous variables are presented as mean ± standard deviation (M ± SD) or median (interquartile range) depending on the normality of the distribution. Comparisons between two groups were performed using Student’s *t*-test for continuous variables or the Mann–Whitney U test for continuous non-parametric variables. The ANOVA, or Analysis of Variance, test was used to determine differences between research results from three or more unrelated samples or groups. The Statistical Package for the Social Sciences (SPSS 24, Chicago, IL, USA) was used for data analysis.

## 3. Results

The session program was carried out between February and March 2024, and the number of sessions attended by the group was 4.0 ± 0.8 out of 6. The mean age among the 18 patients who attended and completed the initial and final questionnaires was 35.8 ± 9.0 years old, with 13 (72%) female patients. The NYHA functional class was 1.89 ± 0.6, and the number of cardiac surgeries carried out during their lifetime was 2.1 ± 0.9. In relation to cardiovascular risk factors, two (11%) patients had arterial hypertension, four (22%) dyslipidemia, one (6%) was a smoker, and five (28%) patients had pacemakers or implantable cardioverter defibrillators. No patient reported the consumption of illicit drugs or alcohol abuse. In relation to psychiatric pathology, five (28%) patients referred to having a previous history of mental problems and six (33%) were under anxiolytic and antidepressant treatment. According to complexity, five (28%) patients had mild defects, six (33%) moderate defects, and seven (38%) great CHD defects (Table 2 shows the different types of CHD included in this study). Regarding employment status, four patients were employed, three retired, one stay-at-home, three were students, one was unemployed, and three patients reported others status. In relation to educational level, 1 patient had elementary education, 1 primary education, 12 secondary education, and 4 university studies.

Table 3 shows the results of the EQ-5D questionnaire (mobility, safe care, usual activities, pain/discomfort, anxiety/depression, and the health scale), the Global Assessment of Functioning (GAF) questionnaire, the Rosenberg Self-Esteem Scale (RSES), the State–Trait Anxiety Inventory (STAI) with the STAI-State and STAI-Trait subscales, and the Beck Depression Inventory-II (BDI-II) at the beginning (pre) and end (post) of the sessions program in the 18 patients with CHD and anxious–depressive symptoms. As can be seen in it, patients with CHD scored higher in the Euro quality of life visual analogue scale and lower in the BDI-II inventory at the end compared to the beginning of the program, which entails a better health condition and lower depressive symptoms at the end of the study. Meanwhile, the Rosenberg Self-Esteem Scale score was close to reaching statistical significance (25.1 ± 5.4 vs. 27.4 ± 6.0, *p* = 0.051).

Meanwhile, when patients with CHD were studied according to gender or their cardiac complexity (mild, moderate, or great defects), no significant score differences were seen in the two subscales of the anxiety questionnaire and the Beck Depression survey before and after the psychological intervention. Similarly, no significant differences were found according to the educational level before and after interventional therapy. Regarding work activity, although no differences were found in the anxiety and depression scores after the psychological intervention, it was observed before the beginning of the study that retired patients scored significantly lower in the trait anxiety questionnaire (STAI-T) than the rest of the patients (23.7 ± 3.7 vs. 31.0 ± 3.1, *p* = 0.033).

Finally, Table 4 shows the satisfaction survey carried out at the end of the sessions program. In it may be seen that participants scored high (on a scale of 0 to 3) in questions such as feeling comfortable with their colleagues (2.5 ± 0.6), recommending the program to other patients (2.3 ± 0.6), or being willing to attend future sessions (2.6 ± 0.8). On the contrary, they had little interest in participating in the online activities and completing the recommended homework.

## 4. Discussion

Improvements in surgical techniques, medical treatments, and overall cardiac care have significantly increased survival rates for individuals with CHD, with many of them reaching into adulthood. In these patients, mental illness encompasses a wide range of mental health conditions which involve changes in emotion, thinking, behavior, and social/work/family networking. Factors such as overprotection, traumatic experiences, multiple hospital admissions, devices’ implantation, heart failure, arrhythmia, endocarditis, catheter or surgical reinterventions, organ dysfunction, and an elevated risk of premature mortality can make these patients more likely to suffer from mental illnesses. In fact, existing data show that depression, anxiety, bipolar disorder, psychosis, attention deficit hyperactivity disorder (ADHD), and autism spectrum disorders occur more often in people with CHD than in healthy counterparts [39]. In this context, a recent study caried out in 15 countries with 3815 participants (age 34.8 ± 12.9 years; 52.7% female) evidenced that in adults with CHD, almost one-third reported symptoms of depression and/or anxiety [40], which is in accordance with the prevalence seen in our population [13]. 

Group therapy is a form of psychotherapy, which involves one or more therapists, that aims to help people manage mental health conditions and cope with negative experiences and behavior. The group favors interaction with others who may be going through similar challenges, sharing valuable feedback at a lower economic and personnel cost than individual therapy. Moreover, it has been shown that group therapy in the general population is as effective as individual therapy for a wide range of metal illnesses such as anxiety [41] and depression [42], relieving the appearance of symptoms. However, the disadvantage is that many people may drop out of the group therapy program [43].

In our series, no significant differences were obtained in the anxiety (STAI) questionnaire, but there were in the Euro quality of life visual analogue scale and the Depression Inventory (BDI-II) after six sessions of group therapy, as also seen by others, in a modest way, using the Hospital Anxiety and Depression Scale (HADS) [42]. Depression and anxiety, while often related and sometimes co-occurring, are distinct conditions with different underlying mechanisms, symptoms, and treatment responses. Therefore, there are several reasons that could explain why our patients with CHD experienced an improvement in depression but not in anxiety symptoms: Firstly, our CHD patients started with low levels of anxiety and light–moderate levels of depression, according to the scales used, which makes clinical improvement more complicated in the anxiety scores. Secondly, anxiety disorders seen in patients with CHD often coexist with other conditions, such as obsessive–compulsive disorder, post-traumatic stress disorder, or specific phobias that may complicate treatment and make anxiety harder to alleviate. Thirdly, stressors that contribute to anxiety, such as ongoing work stress, financial issues, relationship problems, mobbing, or even bullying might be more difficult to manage or change compared to factors contributing to depression. Finally, lifestyle changes that benefit depression, like exercise or improved sleep, might not be as effective in reducing anxiety if the underlying stressors remain.

Similarly, individuals with CHD often face challenges that may affect their self-esteem. However, with appropriate psychological support, education, and family involvement, it is possible to enhance their self-worth and overall well-being. In our series, we saw and increased score in the Rosenberg Self-Esteem Scale (RSES) after the 6-week session period (25.1 ± 5.4 vs. 27.4 ± 6.0, *p* = 0.051) that remained on the margin of significance, reflecting the beneficial effect that group sessions may have not only in reducing depressive symptoms but also in improving self-esteem in this type of patients. As self-esteem and depression are closely linked, with each potentially influencing the other, understanding their relationship seems to be crucial for developing effective interventions to improve mental health outcomes.

In relation to group psychological therapy among patients with CHD, Kovacs et al. [24] had previously confirmed the feasibility of conducting a psychosocial intervention on these patients, although they recommended addressing barriers associated with in-person attendance and the identification of strategies to reduce it. In this context and to improve participants’ adherence, several key strategies should be considered: (a) setting clear and achievable goals, (b) establishing a routine, (c) providing accessible resources, like instructional videos or handouts, (d) regular check-ins, (e) creating a supportive peer environment to share experiences, (f) implementing incentives for consistent participation, (g) educating participants on the benefits of these activities to reinforce their importance, (h) having flexibility in how and when exercises are completed, and, finally, (i) celebrating progress, no matter how small [44,45]. By integrating these strategies, patients may effectively improve adherence to the program, ultimately enhancing participant outcomes.

In this regard, before starting our study, we looked for patients who were committed to the program. We also called by phone and/or sent reminder emails before each of the sessions. Likewise, the reference hospital was equidistant from where most of the patients lived (less than 45 min by car), sessions were carried out in a large and comfortable room to facilitate the relationship with the rest of patients, and those who requested it were provided with confirmation of attending the hospital activity. In fact, only 3 patients out of 21 (14%) did not complete the program. Moreover, our CHD patients scored high in questions related to feeling comfortable with others and meeting their expectations in the satisfaction questionnaire, which meant that most of them would like to repeat the program as well as recommend it to other patients.

## 5. Study Limitations

The lack of significant differences in mobility, safe care, usual activities, pain/discomfort, anxiety/depression, as measured by the EQ-5D questionnaire, and the Global Assessment of Functioning (GAF) score following the six-session psychological program may stem from several interrelated factors. Firstly, the relatively short duration and limited frequency of the intervention may not have been sufficient to elicit meaningful changes in these complex constructs, particularly in a population with chronic health conditions that may require more intensive or prolonged support. Additionally, the inherent variability in individual responses to psychological interventions could mean that some participants may not have engaged with the program content in a way that facilitated change, potentially due to pre-existing psychological resilience or varying levels of motivation. Furthermore, the measurement tools employed may not have been sensitive enough to detect subtle changes in functioning or well-being, particularly in a clinical population where baseline levels of anxiety and functioning may already be skewed. Lastly, external factors such as ongoing medical treatments, social support systems, or life stressors outside the program may have influenced participants’ overall health and well-being, overshadowing any potential benefits derived from the psychological intervention.

Also, limits to generalizability include a single-center study, the low number of patients, and the lack of a randomized design with the inclusion of a control group. However, in our group psychological program, we opted not to include a control group or randomization of samples primarily due to the nature of the intervention and the specific population we aimed to serve. Our program was designed to address urgent mental health needs in a community where access to resources was limited, making it impractical to withhold treatment from a control group. Additionally, the focus was on real-world applicability and immediate support rather than strict experimental conditions. By prioritizing the well-being of participants and the urgency of their needs, we aimed to provide a meaningful intervention that could be implemented in similar settings, even if it meant sacrificing some methodological rigor typically associated with randomized controlled trials. Nonetheless, unlike other studies [28], we had a similar proportion of patients with mild, moderate, and great CHD complexity, and the education level and employment activity were like those of our general population, which makes the results reproducible. Also, most of our patients with CHD had mild depressive symptoms, as also occur in the general population, where mild depression tends to be more common than severe depression [46]. Given the great heterogeneity of CHD, more studies should be carried out with a larger number of patients to verify the usefulness of these programs in this population. Thus, a multifaceted approach that includes longer intervention periods, tailored content, and more sensitive measurement tools may be necessary to better capture the impact of psychological programs in this unique patient population. 

## 6. Conclusions

In conclusion, group therapy was useful to reduce depressive symptomatology among patients with CHD, providing a safe environment to practice social skills and improve relationships, which are often affected by depression. Exposure to different viewpoints and coping strategies seems to help managing depression at a lower cost than individual therapy, which makes it more accessible and of application at a health level.

## Figures and Tables

**Table 1:** Weekly sessions in the adult congenital heart disease (CHD) psychological therapy program.

Weekly Sessions	Program	Time Allocation
Week 1	Description of CHD: Overview of CHD patients, physical limitations, unique aspects, and common dilemmas.	30 min
	Stress Definition: Acute/chronic stress, physiological/cognitive responses, and coping styles.	30 min
	Relaxation Training: Jacobson’s Progressive Muscle Relaxation.	30 min
	*Homework: Recording stressful situations.*	
Week 2	Mind–Body Connection: How emotions affect us, identifying emotions, thoughts, and beliefs about CHD.	30 min
	Cognitive Restructuring: Core beliefs about pathology, ABC model, and cognitive distortions.	30 min
	Mindfulness Training: Conscious thinking and mindfulness techniques.	30 min
	*Homework: Thought recording.*	
Week 3	Events Related to CHD: Workplace harassment, suicidal ideation, and enjoyable activities.	30 min
	Behavioral Activation: Planning simple, realistic activities.	30 min
	Relaxation Training and Mindfulness Training: Jacobson’s and mindfulness techniques.	30 min (15 min each)
	*Homework: List of pleasurable activities and involvement in any of them.*	
Week 4	Physical Activity and Sport: Body responses, erroneous beliefs, and guidelines for safe practice.	30 min
	Diet and Nutrition: Dietary considerations, erroneous beliefs, and a sample weekly nutritional plan.	30 min
	Relaxation and Mindfulness Training: Jacobson’s and mindfulness techniques.	30 min (15 min each)
	*Homework: Sports activity log and weekly diet log.*	
Week 5	Social Skills: Communication, managing relationships, conflict resolution, and building support networks.	30 min
	Role-playing: Practicing conflict resolution in social situations.	30 min
	Relaxation and Mindfulness Training: Jacobson’s and mindfulness techniques.	30 min (15 min each)
	Homework: Recording conflictive dynamics and how to resolve them appropriately.	
Week 6	Program Review: Summary of areas worked on.	45 min
	Closure: Reflection on feelings and program rating.	45 min

**Table 2:** Congenital heart disease (CHD) classification according to the anatomical complexity.

Types of CHD According to Complexity	Number of Patients
**Simple complexity**	5
Atrial septal defect	1
Ventricular septal defect	2
Other simple defects	2
**Moderate complexity**	6
Tetralogy of Fallot	4
Atrioventricular septal defects	2
**Great complexity**	7
Dextro transposition of the great arteries	3
Levo transposition of the great arteries	1
Pulmonary atresia	2
Tricuspid atresia	1
**Total**	18

**Table 3:** Questionnaires before and after the sessions program in patients with congenital heart disease and anxiety/depression symptoms.

	Pre-Program	Post-Program	*p* *
Number of patients	18 (100)	18 (100)	
EQ-5D questionnaire, score			
Mobility	0.11± 0.3	0.11 ± 0.3	1.000
Safe care	0.06 ± 0.2	0.00 ± 0.0	0.331
Usual activities	0.44 ± 0.5	0.28 ± 0.5	0.083
Pain/discomfort	0.5 ± 0.7	0.33 ± 0.6	0.269
Anxiety/Depression	0.83 ± 0.6	0.61 ± 0.6	0.104
Health Scale	7.14 ± 1.6	7.83 ± 1.4	0.012
Global Assessment of Functioning (GAF), score	73 ± 12.8	75 ± 14.7	0.540
Rosenberg Self-Esteem Scale (RSES), score	25.1 ± 5.4	27.4 ± 6.0	0.051
State–Trait Anxiety Inventory (STAI), score			
STAI-State	24.3 ± 5.2	25.9 ± 4.1	0.165
STAI-Trait	31.0 ± 6.3	31.4 ± 3.2	0.726
Beck Depression Inventory-II (BDI-II), score	18.1 ± 12.1	12.3 ± 10.9	0.003

Scores: EQ-5D questionnaire scores from 0 to 5, health scale scores from 0 to 10, Global Assessment of Functioning (GAF) scores from 0 to 100, and Rosenberg Self-Esteem Scale (RSES) scores from 1 to 4 after reverse-scoring the negatively worded items. State–Trait Anxiety Inventory (STAI) scores from 20 to 80 and Beck Depression Inventory-II (BDI-II) scores from 0 to 63. The data are expressed as mean and standard deviation (M ± SD) and as number and percentage. * Continuous data with normal distribution are compared by Student’s *t*-test.

**Table 4:** Satisfaction questionnaire carried out at the end of the sessions program.

	Score
1. Connections to the recommended online activities	0.9 ± 0.6
2. Completion of recommended exercises at home	1.1 ± 0.6
3. Appropriateness of the number of sessions	2.2 ± 0.6
4. Adequacy of patient numbers and time dedicated	1.9 ± 0.7
5. Satisfaction with session topics	2.1 ± 0.6
6. Comfort with other colleagues	2.5 ± 0.6
7. Program meeting your expectations	2.2 ± 0.7
8. Usefulness of what you learned in managing anxiety and depression	1.9 ± 0.6
9. Likelihood of recommending the program to others	2.3 ± 0.6
10. Interest in repeating the program later	2.6 ± 0.8

The first two questions are scored from 0 to 3 as never, ever, frequently, or always. The rest of the questions from 0 to 3 as not suitable at all, partially suitable, adequate, or very suitable. The data are expressed as mean and standard deviation (M ± SD).

## Data Availability

The participants of this study did not give written consent for their data to be shared publicly, so due to the sensitive nature of the research, supporting data are not available.
